# Multi-Modal Imaging to Assess the Interaction Between Inflammation and Bone Damage Progression in Inflammatory Arthritis

**DOI:** 10.3389/fmed.2020.545097

**Published:** 2020-09-25

**Authors:** Justin J. Tse, Scott C. Brunet, Peter Salat, Glen S. Hazlewood, Cheryl Barnabe, Sarah L. Manske

**Affiliations:** ^1^Department of Radiology, Cumming School of Medicine, University of Calgary, Calgary, AB, Canada; ^2^Cumming School of Medicine, McCaig Institute for Bone and Joint Health, University of Calgary, Calgary, AB, Canada; ^3^Biomedical Engineering Graduate Program, Schulich School of Engineering, University of Calgary, Calgary, AB, Canada; ^4^Division of Rheumatology, Department of Medicine, Cumming School of Medicine, University of Calgary, Calgary, AB, Canada

**Keywords:** high resolution peripheral quantitative computed tomography (HR-pQCT), magnetic resonance imaging, multi-modal imaging, image registration, rheumatoid arthritis, subclinical inflammation

## Abstract

Combining results from multiple imaging techniques (i.e., multi-modal imaging) through image registration can result in the better characterization of joint tissue characteristics. In the context of inflammatory arthritis conditions, high-resolution peripheral quantitative computed tomography (HR-pQCT) provides excellent bone contrast while magnetic resonance imaging (MRI) provides superior contrast and resolution of soft tissue and inflammatory characteristics. Superimposing these imaging results upon each other provides a robust characterization of the joint. In a preliminary study of nine rheumatoid arthritis (RA) participants in clinical remission, we acquired HR-pQCT and MR images of their 2nd and 3rd metacarpophalangeal (MCP) joints at two timepoints 6 months apart. We present the benefits of a multi-modal imaging approach, in which we demonstrate the ability to localize regions of inflammation with subtle changes in bone erosion volume. Using HR-pQCT and MRI to visualize bone damage and inflammation, respectively, will improve our understanding of the impact that subclinical inflammation has on bone damage progression, and demonstrating if bone repair occurs where inflammation is resolved. The presented multi-modal imaging technique has the potential to study the progression of bone damage in relation to inflammation that otherwise would not be possible with either imaging technique alone. The multi-modal image registration technique will be helpful to understanding the development and pathogenesis of RA-associated bone erosions. Additionally, multi-modal imaging may provide a technique to probe the tissue-level changes that occur as a result of treatment regimes.

## Multi-Modal Imaging: Utilizing Each Individual Imaging Modality's Strengths

Inflammatory arthritis conditions are complex in pathophysiology and affect many joint tissues. Imaging plays an important role in the diagnosis, treatment evaluation and understanding of pathophysiological processes. Every imaging modality has its own strengths and weaknesses for tissue and disease assessment, due to their differing abilities to provide tissue contrast, spatial resolution, and access to joints of interest. In the context of inflammatory arthritis, conventional radiography provides 2-dimensional (2D) planar, projection images of structural damage (i.e., joint space narrowing, erosions, and osteophytes) of bony features. Computed tomography (CT) provides 3-dimensional (3D) views, resulting in greater contrast and spatial resolution to visualize the same bony features; however, this is at the expense of greater radiation exposure depending on the joints of interest and their proximity to radiation-sensitive tissues and organs. High-resolution peripheral quantitative computed tomography (HR-pQCT) is a CT modality adapted for imaging extremities and provides significantly enhanced contrast, superior spatial resolution of bony features, and reduced radiation dose when compared to conventional whole-body CT. While originally introduced to image the distal tibia and distal radius, applications of HR-pQCT have extended to include the hand, wrist and knee joints (1–3). HR-pQCT allows for the resolution of individual trabeculae (4), and the ability to quantitatively evaluate joint properties. Semi-automated techniques have been developed to assess periarticular bone mineral density and microarchitecture, joint space width as well as erosion volume (5–8). An overview of these techniques is provided in a review by Klose-Jensen et al. (9) in this special issue.

In contrast to HR-pQCT's excellent spatial resolution of bone, ultrasound and magnetic resonance imaging (MRI) have superior contrast and resolution of soft tissue and can detect features of inflammation. While ultrasound can acquire multiplanar images and facilitate the identification and grading of superficial bony features (10, 11), this imaging technique is limited in its application for analysis below the cortical surface and only provides images several centimeters below the probe (12). However, the presence of a power doppler signal identifies sites of active synovial inflammation without exogenous contrast enhancement. On the other hand, MRI facilitates the 3D visualization of internal features such as bone marrow edema but requires the administration of an intravenous gadolinium contrast agent for the accurate differentiation of inflammatory signals from fluid signals. An additional benefit of MRI is that a semi-quantitative assessment of inflammatory arthritis can be evaluated with MR images using validated scoring systems such as the RA MRI scoring system (RAMRIS) (13, 14) and the psoriatic arthritis MRI scoring system (15).

To date, most multi-modal imaging studies examine each imaging modality independently from one another. However, the 3D multiplanar image acquisition from CT and MRI can facilitate the superimposition of images after accurate image co-registration and transformation, providing complementary information (e.g., bone image from CT, synovitis and inflammation from MRI) to better characterize the entire joint.

## Image Registration

A strategy to visualize and understand bone damage in inflammatory arthritis within a patient is to align and superimpose images acquired from longitudinal time points or multiple imaging modalities using a computational tool known as image registration. We present a brief overview of image registration as a technique that can be used for joint-based imaging on HR-pQCT and MRI datasets.

Image registration is a computational process which iteratively searches for the best alignment based on common information within two or more images. The “best” alignment is defined by metrics such as mutual information or the cross-correlation coefficient (16, 17). The cross-correlation coefficient is typically used when measuring the similarity between image registration of images from the same modality, while mutual information is preferred for multi-modal images. The actual image registration can be performed in a *rigid* or *non-rigid* fashion. *Rigid* image registration aligns the anatomy within the images, without changing the shapes, by a series of rotations and translations. *Rigid* registration is particularly useful when aligning images from the same individual where differences in overall bone shape and size are not expected but the position may differ; acquired either at different times from the same modality, or from multiple modalities at the same time. In contrast, *non-rigid* or *deformable* image registration may allow for local image deformations to facilitate their registration. This technique is beneficial to align images from different individuals to either compare differences in shape or define a common region of interest across multiple individuals, or from the same individual when differences in anatomical shape or size exist between the images.

An initial guess of the translations and rotations is typically required to initialize the registration; this can be accomplished by identifying anatomical landmarks on each image, or by using physical properties of the image such as the center of mass. An optimization algorithm then iteratively searches for the most appropriate transformation (i.e., translations and rotations) to reorient and optimally fit one image onto another image (18), until no further improvement in the alignment metric is detected.

### Assessing Longitudinal Changes and Bone Remodeling With HR-pQCT

As previously mentioned, *rigid* image registration is ideal to align baseline and follow-up images from the same participant on the same imaging modality—ensuring the same region-of-interest is evaluated at both timepoints and improve reproducibility (19, 20). In the context of erosion assessment, image registration allows for a careful comparison of bone changes at the site of the erosion that occur over time (20, 21). Specifically, recent developments have applied image-based bone remodeling algorithms (22) to sensitively assess change in erosion volume over time by aligning, registering, and subtracting baseline from follow-up images (21, 23). *Rigid* image registration is particularly well-suited for follow-up imaging because the gross morphological features remain relatively constant over the time frames typically studied (e.g., 6 months to 2 years), and new structural damage is small relative to the overall size and shape of the bone. However, the main barriers to accurate measures of bone remodeling using this technique are patient motion during the scan time and slow bone remodeling rates, as thickness changes must exceed the true spatial resolution of the scanner at ~100 μm (4). While we have demonstrated good performance of this algorithm in rheumatoid arthritis patients with relatively slow rates of bone changes, performance suffers if significant patient motion is present in the baseline or follow-up images (23).

### Multi-Modal Image Registration

The main advantage of imaging the same individual with a different imaging modality is the ability to simultaneously study different tissue characteristics (i.e., CT for bone and MRI for synovitis and inflammation). As intensity values between imaging modalities represent different tissues, we use mutual information to align images from multiple modalities (24, 25). This technique identifies and matches common clusters of regions between the two images; for example, homogeneous regions of one image will be mapped to a homogeneous region in a second image (17).

## Using HR-pQCT and MRI To Investigate The Role of Inflammation In Bone Damage Progression

### Rationale

Timely, target-driven therapeutic intervention with disease modifying medications is currently an effective mechanism to achieve clinical remission in rheumatoid arthritis (RA). Clinical remission is typically defined by a combination of low tender and swollen joint count, patient reported outcomes, and inflammatory biomarkers in RA patients. However, many patients classified as in clinical remission, with an absence of painful, tender or swollen joints on clinical exam, have evidence of “sub-clinical inflammation” present on MRI or ultrasound (26). Unfortunately, a subset of these patients continue to have worsening radiographic damage scores and progressive disability (27, 28). Cross-sectional evidence of patients in remission with inflammation, visualized on ultrasound, showed an altered trabecular bone mineral density as measured by HR-pQCT when compared with those with no subclinical inflammation (29). While acute inflammation plays an important role in bone repair (30), the significance of sustained subclinical inflammation on bone changes over time is unknown. Therefore, we present a pilot study in which we combined imaging modalities for soft tissue and inflammation (MRI) and bone (HR-pQCT) to determine whether we could provide important pathophysiological information on bone damage progression that is not possible with either imaging modality alone.

### Methods

In a preliminary study of nine RA participants recruited based on physician-classified clinical remission, we acquired HR-pQCT (XtremeCTII, Scanco Medical, Brüttisellen, Switzerland) images of the 2nd and 3rd metacarpophalangeal (MCP) joints and MR images (1.5T Optima MR430s, GE Healthcare) of 2nd to 5th MCP joints of the same hand at baseline and 6-month follow-up. The combination of HR-pQCT and a 6-month follow-up was selected because previous research has demonstrated that bone changes may be detected with HR-pQCT as early as three- to 6-months (31); thus, HR-pQCT would provide the resolution necessary to image these changes. MR sequences included T1-weighted sequence with and without fat saturation using the Dixon method in the coronal and axial plane as well as a short tau inverted recovery (STIR) sequence in the coronal plane, and a repeat of T1-weighted sequences after administration of gadolinium contrast. Approval for all procedures was obtained from the Conjoint Health Research Ethics Board at the University of Calgary (REB17-0188).

Three-dimensional (3D) volumetric joint space width (JSW; volume, minimum, maximum, and mean) was quantified on HR-pQCT images (5). Erosion volume was quantified on HR-pQCT images at baseline and follow-up using the Medical Image Analysis Framework [MIAF, University of Erlangen; (6)]. A radiologist (PS) scored bone marrow edema, synovitis and erosions on MR images using the RAMRIS scoring system (13). A multi-modal image registration technique was adapted and employed to register and transform the MR image to be overlaid with the HR-pQCT image at each timepoint (32) (Supplementary Figure 1). On HR-pQCT images, a trained observer (SB) followed Study grouP for x-trEme Computed Tomography in Rheumatoid Arthritis (SPECTRA) guidelines for the definition of a bone erosion (33): a cortical break observed in two consecutive slices and two orthogonal planes. Bone marrow edema and erosion volume changes were confirmed by visual inspection after superimposition of HR-pQCT and MRI images.

Statistical analysis was completed using R (v3.4.3) in RStudio v1.1.423. To examine group-level time effects, the Wilcoxon rank sum test was used for clinical outcomes and RAMRIS scores, while a non-parametric marginal model for longitudinal designs [nparLD package in R (34)] was used for total erosion volume and JSW. Significance was observed if *p* < 0.05. Changes in erosion volume and JSW at the individual joint level were evaluated against the least significant change (LSC) (35), which was calculated based on previous scan-rescan reproducibility work (36). Using the LSC criteria, participants were classified in one of three groups for each outcome measure: a significant decrease, stable, or a significant increase (20). Results are presented as median and interquartile range (IQR) unless otherwise indicated.

### Results

#### Clinical Outcomes

The nine participants (4 females and 5 males) in clinical remission [DAS28CRP 1.75 (1.63–2.6)], ESR 7 (3–11), CRP 3.4 (1.1–4.2), were 62 (59–63) years old, and had been diagnosed with RA for 7.8 (5.1–8.0) years. All participants were being treated with conventional disease-modifying anti-rheumatic drugs (DMARDs; methotrexate, sulfasalazine, and/or hydroxychloroquine), six with biologic DMARDs (3 with adalimumab, 1 golimumab, 1 abatacept, 1 rituximab), 1 with denosumab (a biologic non-DMARD) and 1 with 2.5 mg bi-daily prednisone.

Group level results are presented for those in clinical remission only. Over the 6-month follow-up, there was a significant improvement in patient global assessment (−1 improvement on a 1–10 scale, IQR −2 to 0, *p* = 0.03). There were trends toward further improvements in DAS28 (−0.42 IQR −0.82 to −0.03, *p* = 0.07), Jebsen Taylor Hand Function test z-score (−0.8 IQR −1.2 to −0.3, *p* = 0.08), HAQ score (−0.12, IQR −0.50 to −0.26, *p* = 0.09), the self-reported Disabilities of the Arm, Shoulder, and Hand questionnaire (DASH, −7.5, IQR −11.4 to 0, *p* = 0.08) and pain (−1 improvement on a 1–10 scale, IQR −2 to 0, *p* = 0.06).

#### HR-pQCT Outcomes

The analysis for JSW and erosions was performed on the acquired HR-pQCT of the 2nd and 3rd MCP joints; however, for both analyses, the same two participants were excluded due to motion (4 joints) and 2 additional joints from two separate participants could not be segmented accurately. Therefore, a total of 12 joints were analyzed across 7 participants. At the group level, there were no significant changes over time in any of the joint space outcomes (*p* > 0.05). When compared with the LSC, the joint space parameters remained stable except for one patient who showed a decrease in minimum joint space width (−0.42 mm) in one joint.

Across the 12 joints from 7 participants, 19 erosions were found to range in size from 0.19 mm^3^ to 93.11 mm^3^. Ten erosions were identified at the 2nd MCP, and 9 erosions identified at the 3rd MCP. At the group level, there were no significant changes in the total erosion volume per joint (*p* = 0.11). When compared against the LSC, 5 patients had erosion volumes that stayed stable in 10 joints, one patient had a decrease in total erosion volume (−2.3 mm^3^) in one joint, and one participant had an increase in erosion volume (+2.1 mm^3^) in one joint. These changes can be visualized with longitudinal image registration (**Figure 2**).

#### MRI Outcomes

The RAMRIS total score across MCP joints 2–5 for all 9 patients in clinical remission was 3.0 (1–6) for synovitis, 2.0 (1–2) for bone marrow edema, and 2 (2–4) for erosions at baseline. At follow-up the mean total scores were 5 (3–6) for synovitis, 2 (1–5) for bone marrow edema, and 3 (2–6) for erosions. There were no significant differences (*p* > 0.05) in RAMRIS scores between baseline and follow-up for synovitis, edema, or erosions.

When looking at the 2nd and 3rd MCPs only, at baseline, 1 joint had no evidence of synovitis while 9 joints had a synovitis score of 1 (mild), and 8 joints had a synovitis score of 2 (moderate). Over the 6-month period, 9 joints were scored as stable, 5 joints had an increase of 1, 1 joint had an increase of 2, and 3 joints had a decrease in synovitis.

For bone edema at baseline in the 2nd and 3rd MCPs, 10 joints had no edema at either the proximal phalange or distal metacarpal, and 8 joints had an edema score of 1 (which represents a volume of bone infiltration between 1 and 33% by the edema). Over the 6-month period, 11 joints had stable bone edema status, while 5 joints showed an increase in edema, and 2 joints showed a decrease in edema.

For the baseline erosions scored on MRI, 9 participants had evidence of erosions on the 2nd MCP. Eight of these had an erosion score of 1 (1–10% of bone covered by erosion) and one bone had an erosion score of 2 (11–20% bone covered by erosion). For the 3rd MCP, 4 participants had evidence of erosions with 3 having a score of 1, and 1 participant having a score of 2. Over the 6-month period, 12 joints had stable erosion scores on MRI while 2 joints showed decreases and 4 joints showed increases.

#### Relationship Between MRI and HR-pQCT Outcomes

One participant with no evidence of bone marrow edema or synovitis near the site of the erosion underwent a significant decrease in erosion volume, assessed by HR-pQCT, over the 6-month follow-up. This was also consistent with a decrease in MRI erosion score. In contrast, all participants who presented with MRI-evidence of inflammation within the joints maintained no change in erosion volume over the 6-month follow-up, similarly to those in clinical remission.

**Figure 1:**
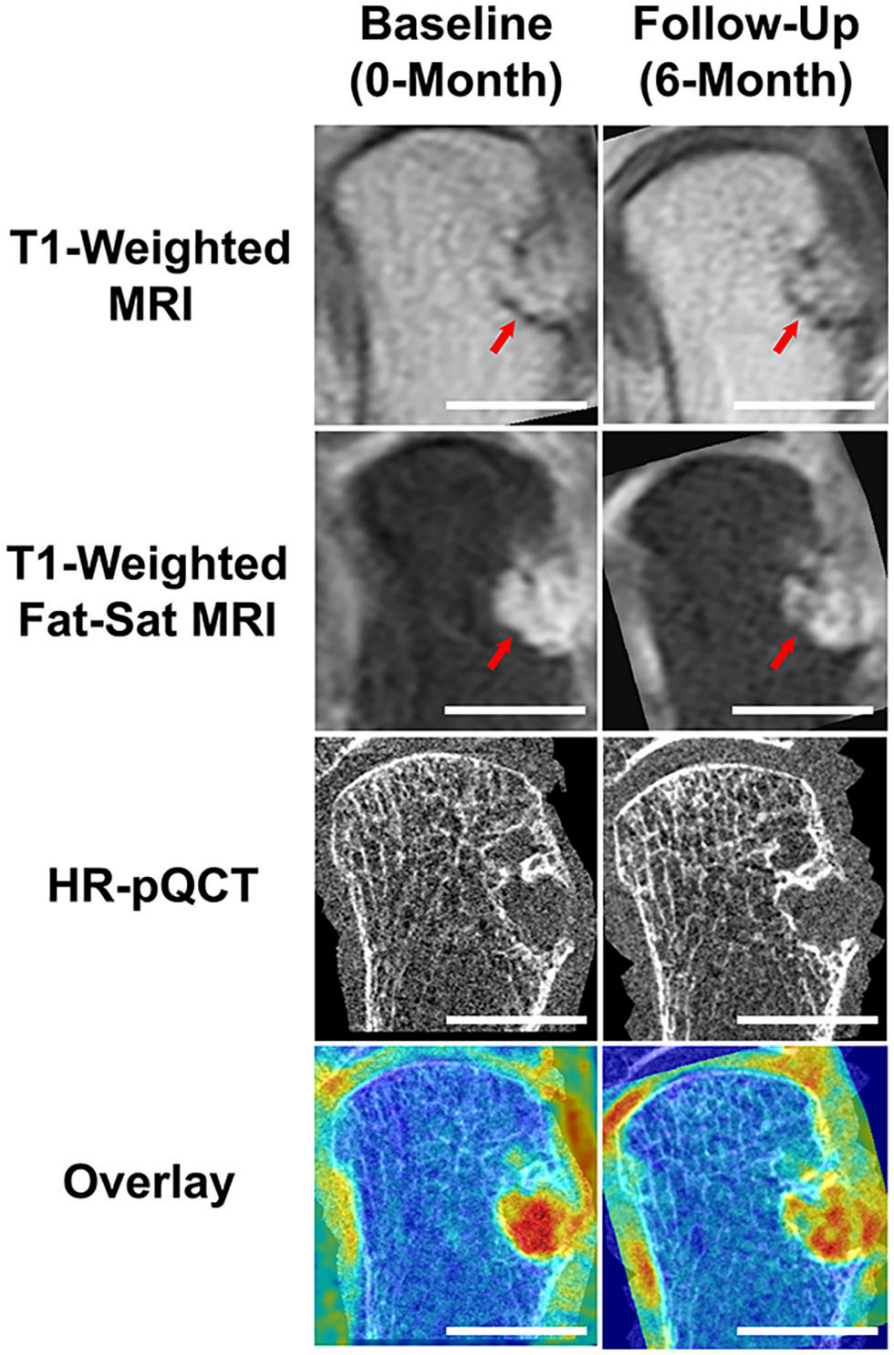
Results demonstrating the benefits of multi-modal imaging. Using MRI and RAMRIS scoring, this RA participant was noted to have a dramatic increase in bone erosion score during their 6-month follow-up, when compared to their baseline score. The post-contrast (Gd) T1-weighted MRI visually displays a larger erosion in the follow-up image (note red arrows). Through accurate image co-registration and overlay, we can observe that the increase in bone erosion score may be due to the differences in positioning during acquisition. While the post-contrast, fat-saturated T1-weighted MRIs display a difference in intensities, the HR-pQCT demonstrates an almost identical erosion size. The Overlay demonstrates the addition of the T1-weighted MRI overlaid with the respective, co-registered HR-pQCT scans. Scalebars represent 1 cm.

At the group level, baseline bone marrow edema was not associated with increased erosion volume, however the participant with the largest increase in erosion volume also demonstrated progression in the bone marrow edema score. In addition, more participants had changes in erosion severity when evaluated by MRI than by HR-pQCT (Figure 1).

**Figure 2:**
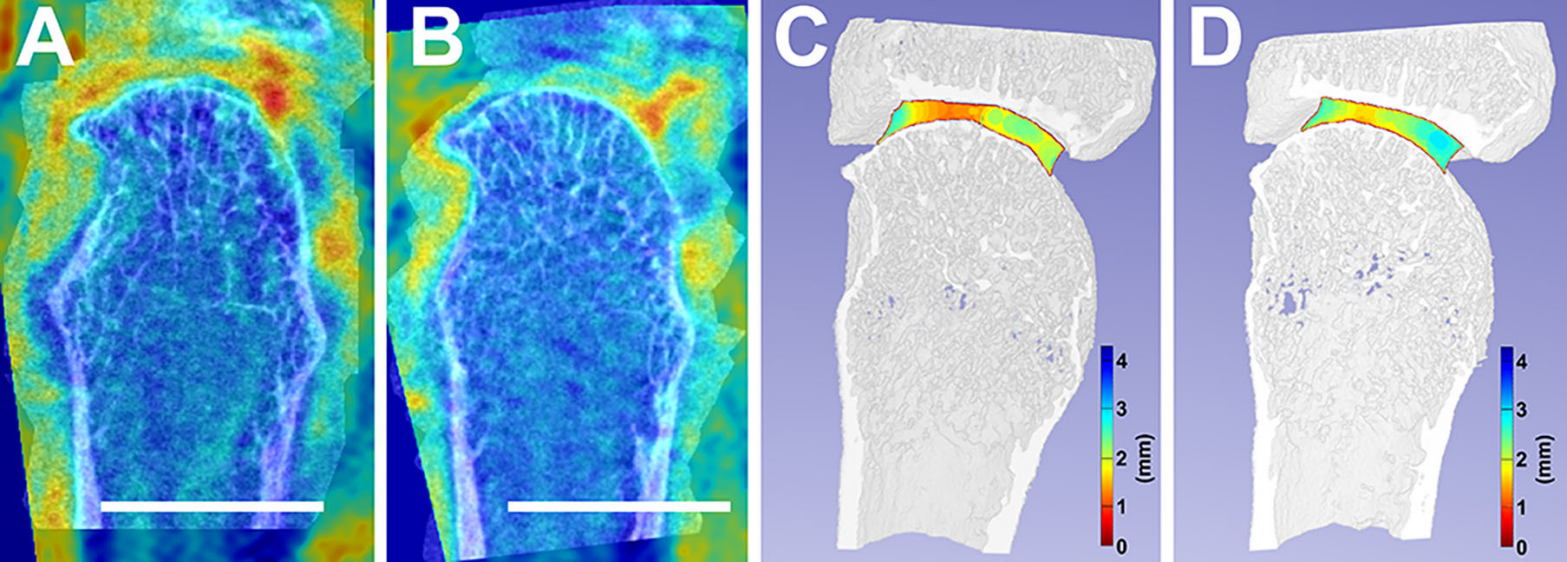
Accurate image co-registration between a selected individual's baseline, 0-month **(A)**, and follow-up, 6-month **(B)** facilitated the overlay between their MRI and HR-pQCT. This image co-registration allows for the consistent analysis of the same areas-of-interest between timepoints. Additionally, joint space width changes from baseline **(C)** to follow-up **(D)** are compared. White scalebars represent 1 cm.

One participant had a significant decrease in minimum JSW (Figure 2). This participant had a synovitis score of 2 at baseline which decreased over 6-months, but also demonstrated a decrease in ESR, a moderate EULAR DAS28 response, and a large decrease in HAQ and pain.

## Discussion

We have presented the benefits of a multi-modal imaging approach through our ability to now localize how synovitis and bone marrow edema affect bone damage in RA patients. This was achieved by using accurate image registration, to facilitate improved reproducibility by adjusting for differences in positioning between scans and accurately mapping soft tissues and inflammation onto bone. In addition, we observed discordances in erosion changes assessed by HR-pQCT and MRI. The higher spatial resolution of the HR-pQCT images, combined with longitudinal registration allowed us to detect bone changes with greater sensitivity and reproducibility (19, 37, 38)—emphasizing the utility of multi-modal imaging approaches to fully understand all the tissue types involved in the progression of a disease.

While we have shown the utility of HR-pQCT for research purposes, its applicability as routine imaging in clinical studies remains unknown. Motion artifact due to patient motion during acquisitions can result in missing data. While we implemented an overlapping stacking process, which significantly reduced the need to discard scans due to motion artifact, rather than eliminating the artifact, this process simply smoothed the data (36). Currently, we have begun exploring implementing established motion correction algorithms that will increase the usability of acquired data (39, 40). Additionally, recent research has shown that the current SPECTRA defined bone erosion definition may result in the under-estimation of the number of bone erosions, when compared to histological evaluation (41). It should be noted that while we propose the implementation of HR-pQCT to measure disease progress in the research setting, the extra scanning will unavoidably increase radiation dose to the patient; however, a single HR-pQCT scan is < 20 μSv, which equates to a third of the radiation dose one would receive on a transcontinental flight. Regardless, the impact of the multi-modal imaging and in-depth erosion evaluation measures on clinical outcomes needs to be determined. Thus, a larger sample size, further in-depth analysis, and multiple reader assessments will be required to better understand how individual erosions changed over the time points.

Despite these limitations, the finding of more erosion changes in RAMRIS scores than HR-pQCT was surprising. Two of the joints with erosion progression on MRI had an erosion that was identified at follow-up but not baseline on MRI; the same erosions were identified but at both times on HR-pQCT. This discrepancy may be the result of differences in positioning on the MRI at baseline and follow-up resulting in the inability to see the small erosion at baseline or the improved sensitivity of HR-pQCT as a tool for erosion identification. Also, there were scans that met our motion scoring criteria, but within the volume of the erosion on HR-pQCT there was motion that appeared to hinder the erosion volume segmentation on HR-pQCT. Finally, an increase of 1 for the RAMRIS erosion equates to only a 10% increase in erosion volume. Despite the increased sensitivity of HR-pQCT, a change of only 10% in the erosion volume would generally not result in a change that exceeds the LSC.

Despite the minimal bone changes observed across the participants over multiple time-points, likely explained by the patients' low disease activity, short follow-up period and sample size, we have presented the benefits of a multi-modal imaging approach that can be utilized to simultaneously visualize, localize, and characterize how synovitis and bone marrow edema may affect bone damage in RA patients. The combination of HR-pQCT and MRI to visualize bone damage and inflammation, respectively, provides the ability to probe mechanistic questions about whether bone loss occurs via an inside-out or outside-in approach (38, 42, 43), as well as how local inflammatory signals lead to bone erosion progression. This multi-modal imaging approach will improve our understanding of the role of both clinical levels of inflammation in arresting bone repair in inflammatory arthritis and the impact that subclinical inflammation has on bone damage progression. In addition, it will allow us to probe the pathophysiological processes that occur at the tissue level in the development and progression of bone damage, and the mechanisms by which treatments halt or repair bone damage, further increasing our understanding of the etiology and pathophysiology of diseases.

Overall, multi-modal imaging can be combined synergistically with functional and biochemical imaging. Examples of other applications include combining CT with positron emission tomography (PET). PET/CT using 2-deoxy-2[18F]fluoro-d-glucose (FDG) to visualize glucose metabolism has been has recently been used to investigate increased glycolysis due to inflammation in inflammatory arthritis, demonstrating correlations between metabolic markers identified with PET and clinical diseases activity in rheumatoid arthritis (44), and between PET activity score and inflammation score on MRI in patients with sacroiliitis in spondylarthropathy (45). Further, PET uptake predicts radiographic progression in RA (46). PET-MRI techniques are also emerging to investigate musculoskeletal disease, which allow linkage of bone metabolic activity with high levels of soft tissue contrast (47, 48), demonstrating that sites of synovitis and tenosynovitis identified on MRI are linked to increased FDG uptake.

Multi-modal imaging enables investigation of underlying tissue pathology using non-invasive techniques. Advances in hardware and software have enabled unprecedented spatial and contrast resolution. Combined with image registration and other image processing techniques, multi-modal imaging provides the great potential to study the mechanisms underlying tissue changes in inflammatory arthritis that otherwise would not be possible with any single imaging technique alone.

## Data Availability Statement

The raw data supporting the conclusions of this article will be made available by the authors, without undue reservation.

## Ethics Statement

The studies involving human participants were reviewed and approved by Conjoint Health Research Ethics Board at the University of Calgary. The patients/participants provided their written informed consent to participate in this study.

## Author Contributions

PS, GH, CB, and SM contributed conception and design of the study. SB, CB, and SM acquired the data. SB and JT performed data analysis. JT and SM drafted the manuscript. All authors contributed to manuscript revision, read, and approved the submitted version.

## Conflict of Interest

The authors declare that the research was conducted in the absence of any commercial or financial relationships that could be construed as a potential conflict of interest.

## Supplementary Material

Supplementary Figure 1 Workflow for MRI-HR-pQCT image registration. **(A)** The T1-weighted MRI and HR-pQCT images are landmarked using common anatomy and the T1-weighted image is registered to the HR-pQCT image space. **(B)** The transformation is then applied to the contrast enhanced fat-saturated MRI image and **(C)** overlaid with the HR-pQCT image. A color map can be applied to visualize areas of inflammation (red) in relationship to the bone damage.

Supplementary Table 1 Overall demographic parameters outlining our participants (top), and changes in their patient- and clinical-reported outcomes after 6-months (bottom). DAS, Disease Activity Score; ESR, Erythrocyte Sedimentation Rate; CRP, C-Reactive Protein; JTHF, Jebsen-Taylor Hand Function Test; HAQ, Health Assessment Questionnaire; DASH, Disabilities of the Arm, Shoulder, and Hand. Values are reported as mean (range) unless reported otherwise [i.e., mean (IQR)].

Supplementary Table 2 Reported outcomes from HR-pQCT and MRI displaying the changes in erosions and joint space width, as calculated from HR-pQCT, and RAMRIS score (i.e., synovitis, bone marrow edema, and erosions) as calculated from MRI. Values are reported as mean (IQR) unless noted otherwise.
