# Supplementary figures and images for: Multi-Modal Imaging to Assess the Interaction Between Inflammation and Bone Damage Progression in Inflammatory Arthritis
Source: Front Med (Lausanne). 2020 Sep 25;7:545097. doi: 10.3389/fmed.2020.545097 (PMC7544988; doi:10.3389/fmed.2020.545097)

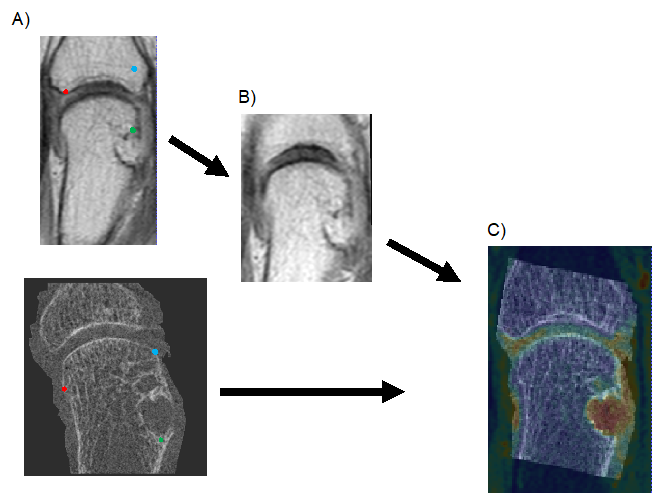

Supplement: Supplementary Figure 1 — Workflow for MRI-HR-pQCT image registration. (A) The T1-weighted MRI and HR-pQCT images are landmarked using common anatomy and the T1-weighted image is registered to the HR-pQCT image space. (B) The transformation is then applied to the contrast enhanced fat-saturated MRI image and (C) overlaid with the HR-pQCT image. A color map can be applied to visualize areas of inflammation (red) in relationship to the bone damage. [file Image_1.TIF]

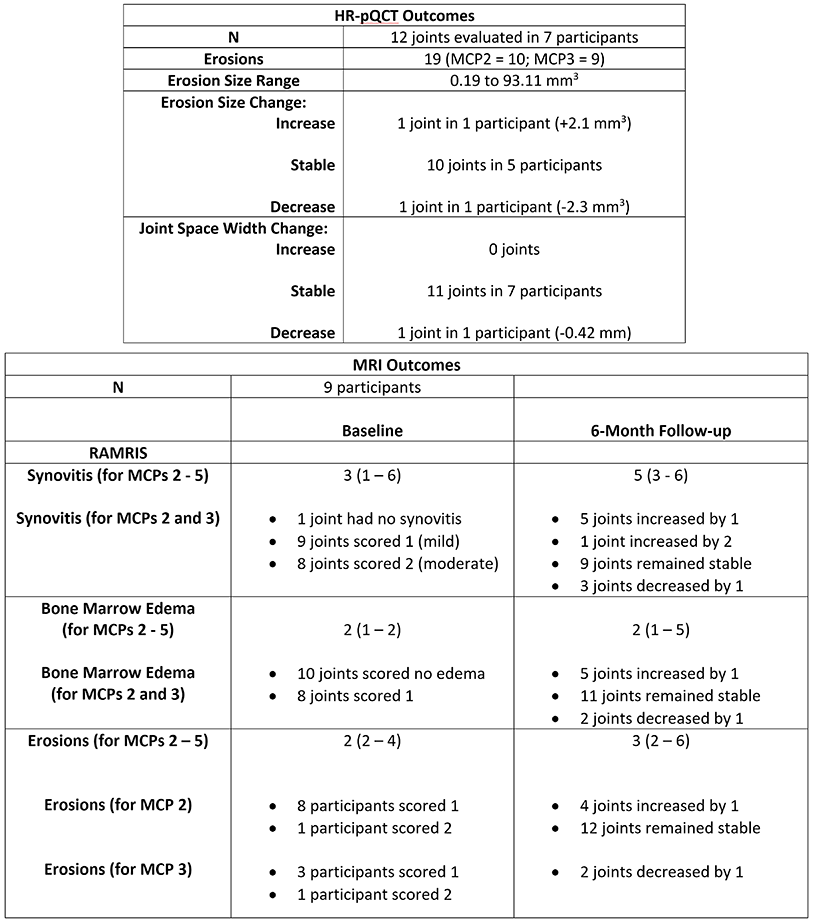

Supplement: Supplementary Table 1 — Overall demographic parameters outlining our participants (top), and changes in their patient- and clinical-reported outcomes after 6-months (bottom). DAS, Disease Activity Score; ESR, Erythrocyte Sedimentation Rate; CRP, C-Reactive Protein; JTHF, Jebsen-Taylor Hand Function Test; HAQ, Health Assessment Questionnaire; DASH, Disabilities of the Arm, Shoulder, and Hand. Values are reported as mean (range) unless reported otherwise [i.e., mean (IQR)]. [file Image_2.TIF]

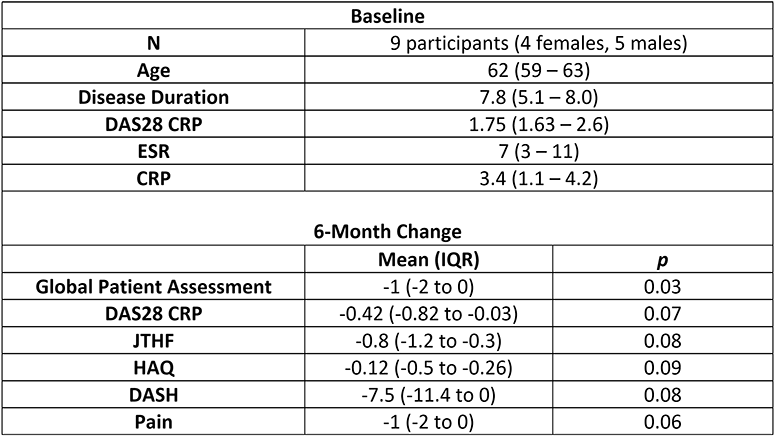

Supplement: Supplementary Table 2 — Reported outcomes from HR-pQCT and MRI displaying the changes in erosions and joint space width, as calculated from HR-pQCT, and RAMRIS score (i.e., synovitis, bone marrow edema, and erosions) as calculated from MRI. Values are reported as mean (IQR) unless noted otherwise. [file Image_3.TIF]
